# Long-term outcomes after severe acute kidney injury in critically ill patients: the SALTO study

**DOI:** 10.1186/s13613-023-01108-x

**Published:** 2023-03-13

**Authors:** Khalil Chaïbi, Franck Ehooman, Bertrand Pons, Laurent Martin-Lefevre, Eric Boulet, Alexandre Boyer, Guillaume Chevrel, Nicolas Lerolle, Dorothée Carpentier, Nicolas de Prost, Alexandre Lautrette, Anne Bretagnol, Julien Mayaux, Saad Nseir, Bruno Megarbane, Marina Thirion, Jean-Marie Forel, Julien Maizel, Hodane Yonis, Philippe Markowicz, Guillaume Thiery, Frédérique Schortgen, Cécile Couchoud, Didier Dreyfuss, Stephane Gaudry

**Affiliations:** 1 Present Address: Service de Réanimation Médico-Chirurgicale, AP-HP, Hôpital Avicenne, 125 rue de Stalingrad, 93000 Bobigny, France; 2 UMR_S1155, French National Institute of Health and Medical Research (INSERM), CORAKID, Hôpital Tenon, Sorbonne Université, 75020 Paris, France; 3 Service Anesthésie Réanimation Hôpital Privé Claude Gallien, Quincy-Sous-Sénart, France; 4 Service de Réanimation, CHU de Pointe à Pitre-Abymes, CHU de la Guadeloupe, France; 5 Réanimation médico-chirurgicale, CHG, La Roche-sur-Yon, France; 6 Réanimation polyvalente, CH René Dubos, Pontoise, France; 7 Réanimation médicale CHU Bordeaux, Hôpital Pellegrin, Bordeaux, France; 8 Service de réanimation, Centre Hospitalier Sud Francilien, Corbeil Essonne, France; 9 Département de réanimation médicale et médecine hyperbare, CHU Angers, Universitéd’Angers, Angers, France; 10 Réanimation médicale, CHU Rouen, Rouen, France; 11 Assistance Publique-Hôpitaux de Paris, Hôpitaux Universitaires Henri Mondor, DHU A-TVB, Service de réanimation médicale, Créteil, France; 12 CARMAS research group and UPEC-Université Paris-Est Créteil Val de Marne, Créteil, France; 13 Réanimation médicale, Hôpital Gabriel Montpied, CHU de Clermont-Ferrand, Clermont- Ferrand, France; 14 Réanimation médico-chirurgicale, Hôpital de La Source, Centre Hospitalier Régional d’Orléans, BP 6709, 45067 Orléans Cedex, France; 15 Service de Pneumologie et Réanimation Médicale, APHP, Groupe Hospitalier Pitié-Salpêtrière, Paris, France; 16 Centre de Réanimation, Faculté de Médecine, CHU de Lille, Université de Lille, 59000 Lille, France; 17 Réanimation Médicale et Toxicologique, Hôpital Lariboisière, INSERM U1144, Université Paris-Diderot, Paris, France; 18 Réanimation polyvalente, CH Victor Dupouy, 95107 Argenteuil Cedex, France; 19 Service de réanimation des Détresses respiratoires aiguës et infections sévères, Hôpital Nord Marseille, Marseille, France; 20 Service de réanimation médicale INSERM U1088, Centre hospitalier universitaire de picardie, Amiens, France; 21 Réanimation médicale, Hôpital de la Croix Rousse, Lyon, France; 22 Réanimation, CH, Cholet, France; 23 Réanimation médicale, CHU Saint Etienne, 42270 Saint Priest en Jarez, France; 24 Centre Hospitalier Intercommunal, Service de Réanimation Polyvalente Adulte, Créteil, France; 25 REIN registry, Agence de la biomédecine, Saint Denis La Plaine, France; 26 Service de Médecine Intensive Réanimation, Hôpital Louis Mourier, Assistance Publique Hôpitaux de Paris, Paris, France; 27 Université Paris-Cité, Paris, France

**Keywords:** Acute kidney injury, Renal replacement therapy, Long-term outcomes, Worsening renal failure

## Abstract

**Background:**

The extent of the consequences of an episode of severe acute kidney injury (AKI) on long-term outcome of critically ill patients remain debated. We conducted a prospective follow-up of patients included in a large multicenter clinical trial of renal replacement therapy (RRT) initiation strategy during severe AKI (the Artificial Kidney Initiation in Kidney Injury, AKIKI) to investigate long-term survival, renal outcome and health related quality of life (HRQOL). We also assessed the influence of RRT initiation strategy on these outcomes.

**Results:**

Follow-up of patients extended from 60 days to a median of 3.35 years [interquartile range (IQR), 1.89 to 4.09] after the end of initial study. Of the 619 patients included in the AKIKI trial, 316 survived after 60 days. The overall survival rate at 3 years from inclusion was 39.4% (95% CI 35.4 to 43.4). A total of 46 patients (on the 175 with available data on long-term kidney function) experienced worsening of renal function (WRF) at the time of follow-up [overall incidence of 26%, cumulative incidence at 4 years: 20.6% (CI 95% 13.0 to 28.3)]. Fifteen patients required chronic dialysis (5% of patients who survived after day 90). Among the 226 long-term survivors, 80 (35%) answered the EQ-5D questionnaire. The median index value reported was 0.67 (IQR 0.40 to 1.00) indicating a noticeable alteration of quality of life. Initiation strategy for RRT had no effect on any long-term outcome.

**Conclusion:**

Severe AKI in critically ill patients was associated with a high proportion of death within the first 2 months but less so during long-term follow-up. A quarter of long-term survivors experienced a WRF and suffered from a noticeable impairment of quality of life. Renal replacement therapy initiation strategy was not associated with mortality outcome.

**Graphical Abstract:**

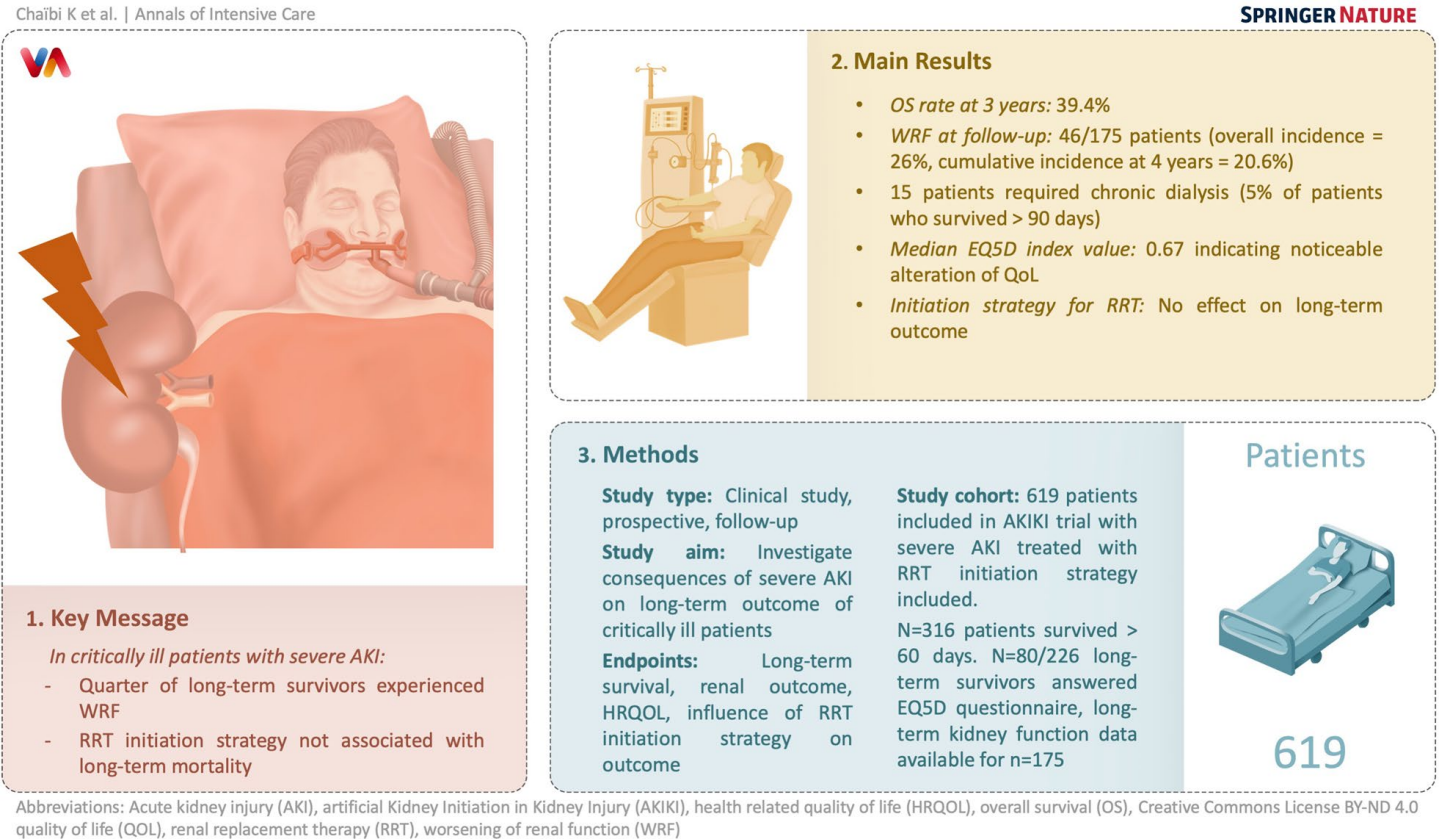

**Supplementary Information:**

The online version contains supplementary material available at 10.1186/s13613-023-01108-x.

## Introduction

Acute kidney injury (AKI) is frequent among critically ill patients and associates with increased morbidity and mortality [1] First considered as a totally reversible syndrome, AKI is now recognized as a risk factor for chronic kidney disease (CKD) [2, 3]. This risk persists even after the normalization of serum creatinine (SCr) level following the acute episode [4, 5]. Patients requiring dialysis for CKD have an altered health related quality of life (HRQOL) and renal replacement therapy (RRT) represents a major cost item for health care systems [6, 8]. In addition, AKI may also impact long-term survival [4, 9, 10]. The majority of studies evaluating long-term outcome after AKI were retrospective and were based on administrative databases and not on actual patient data for a large part [1, 11]. In addition, patient population was markedly heterogeneous in terms of baseline characteristics and of AKI severity [12]. These considerations explain why prospective long-term follow-up of critically ill patients with severe AKI has been considered as a research priority [13, 14]. We took advantage of our large multicenter randomized controlled trial (RCT) on renal replacement therapy initiation strategy for severe AKI (stage 3 of KDIGO classification [15]) in critically ill patients [16] to conduct a long-term follow-up study. The aim of the present study was to investigate long-term survival, renal outcomes and health related quality of life (HRQOL). We hypothesized that some critically ill patients with severe AKI will have poor long-term outcomes. We also aimed to investigate the influence of the RRT initiation strategy on these outcomes because recent large randomized controlled trials on this subject did not assess long-term outcomes.

## Methods

### Study design and patients

The AKIKI trial was an institutionally sponsored, unblinded, prospective, multicenter, open-label, two-group RCT conducted in 31 intensive care units in France from September 2013 through January 2016. Design and methods of the trial have been previously published [17]. Patients with severe AKI, defined by KDIGO stage 3 classification, compatible with the diagnosis of acute tubular necrosis in a context of ischemic or toxic aggression and receiving invasive mechanical ventilation and/or catecholamine infusion (see inclusion criteria in supplementary appendix) were randomly allocated to one of the two following RRT initiation strategies: an early strategy where RRT was initiated within 6 h after AKI KDIGO 3 status was documented; a delayed strategy where RRT was initiated if one or more of the following events occurred: serum potassium concentration greater than 6 mmol/L (or greater than 5.5 mmol/L that persisted despite well-conducted medical treatment), a pH below 7.15 in the context of pure metabolic or mixed acidosis, an acute overload pulmonary edema generating severe hypoxemia, a serum urea concentration higher than 40 mmol /L, oliguria or anuria for more than 72 h.

The duration of the initial follow-up for each patient was 60 days from randomization. For the present study, we extended this follow-up from day 60 and prospectively assessed survival, renal outcomes and HRQOL. Five patients only over a total of 619 in the original study [16] were lost to follow-up. However, we retrieved long-term vital status for 3 through administrative data.

Informed consent was obtained after oral and written delivery of information. Patients who were reached by phone during the long-term follow-up were asked to verbally confirm their consent. The protocol was approved by the ethics committee of the French Society of Intensive Care Medicine and by the appropriate French legal authority (Comité de Protection des Personnes d’Ile de France VI) for all participating centers. Patients or their surrogates were informed that they could decline to participate at any time.

### Data collection

Clinical and biological data were collected each day from randomization in the trial until ICU discharge or until 60 days after randomization in the initial study. For the present study and in order to adequately evaluate kidney function course, we estimated glomerular filtration rate (eGFR) using the Cockcroft formula [18]. We defined CKD by an eGFR < 60 ml/min [19] (Additional file 1: Table S1). We identified all patients who survived 60 days after randomization. We tried to reach all surviving patients (or their surrogate) by phone. During telephone interview, we collected the value for last known Scr concentration, the need for chronic RRT and we assessed HRQOL using the EuroQol Group 5 dimensions tool (EQ-5D-5L) [20]. We first ruled-out any acute event before considering Scr concentration. This allowed exclusion of the influence of a new AKI episode on this value. We also retrieved when available, the nearest non-emergency department SCr concentration obtained 7 to 365 days prior to index hospitalization in order to refine baseline kidney function previously collected in AKIKI. The interviewers were blinded to original study treatment allocation. We attempted to reach the general practitioner (GP) of each surviving patient by phone or email. When neither the patient, surrogate nor GP was available, we requested a certificate of birth from the birthplace townhall (as allowed by French law) that indicates the date in case of death. To avoid missing data on the need for chronic RRT (hemodialysis, peritoneal dialysis or renal transplantation), we queried the French National REIN registry (hosted by French Agency of Biomedicine) which aggregates all patients receiving chronic RRT in France [21].

### Long-term outcomes

- Survival
  Overall survival was assessed at the time of the long-term follow-up (up to 7 years after the ICU stay).
- Renal outcomes
  Because CKD is defined by the persistence of kidney disease for a period of more than 90 days [19], all renal outcomes were assessed beyond this point. They included worsening renal function (WRF) which was defined differently according to CKD status at baseline.
- For non-CKD patients at baseline, WRF was defined by the occurrence of CKD (progression from stage 1 or 2 of KDIGO CKD nomenclature to a stage 3, 4 or 5) [15].
- For CKD patients at baseline, WRF was defined by the progression from a stage 3 to a stage 4 or 5.
  We also assessed the number of patient dependent on chronic dialysis and/or who benefited from kidney transplantation.
- HRQOL

Health-related quality of life was assessed with the EQ-5D questionnaire [22] which consists in evaluation of mobility, self-care, usual activities, pain and anxiety and derives an index value from 0 to 1 where 0 is a health state equivalent to death and 1 the best imaginable state. A negative score indicates a state considered “worse than death” (Additional file 1: Figure S2).

### Statistical analyses

Qualitative variables were compared using a Chi-squared test or a Fisher’s exact test as appropriate, unless otherwise stated. Continuous variables were compared using a Student’s t-test or a Wilcoxon test as appropriate. Where data were missing, we reported the number of available observations and made no assumptions about missing values. We present the results for the overall sample and according to randomization groups (early *versus* delayed RRT initiation strategy).

Survival curves were generated from randomization to long-term follow-up according to the Kaplan–Meier method. The log-rank test was used to compare the 2 groups survival curves.

Univariate and multivariate analysis with Cox model were used to identify risk factors associated with long-term mortality. Variables with *p* < 0.1 in the univariate analysis as well as those that are clinically important like severity, age and some comorbidities were included in the model. Schoenfeld residuals are presented in the supplementary appendix (Additional file 1: Figure S3) to check the proportional hazards assumption. We analyzed risk factors associated with the occurrence of WRF. In this context, we tested variables which have been reported to be potentially associated with worse renal outcomes (CKD, CCF, diabetes, age, hypertension, severity scores) [23]. We also assessed a potential association between RRT initiation strategy and WRF. Since WRF and death are competing events, the use of separate Kaplan–Meier curve and proportional hazard (PH) Cox model (“cause-specific analysis”) for each type of event is inappropriate, because censoring mechanism could not be considered uninformative. In order to appreciate the occurrence of WRF, we calculated the cumulative incidence function (CIF) of each event type, overall and by each factor of interest. The CIF curves represent the cumulative probability of failure from a specific cause over time. The analysis used PH Fine and Gray model which extends the PH Cox model to the presence of competing events. The effect of each factor on each type of event in competition was estimated using the sub-hazards ratio (SHR) and its 95% confidence interval. Interpretation of SHR is similar to that of the Cox model (cause specific) hazard ratio.

We also drew an alluvial diagram showing movement of patients between eGFR categories (see KDIGO CKD nomenclature in Supplementary appendix) between baseline (before severe AKI episode) and long-term follow-up.

All tests were two-sided at a confidence level of 5%. Statistical analyses were conducted using R v.4.1.0 and Prism v 8.4.2. Flowchart was provided with whimsical tool.

## Results

### Study patients

**Fig. 1:**
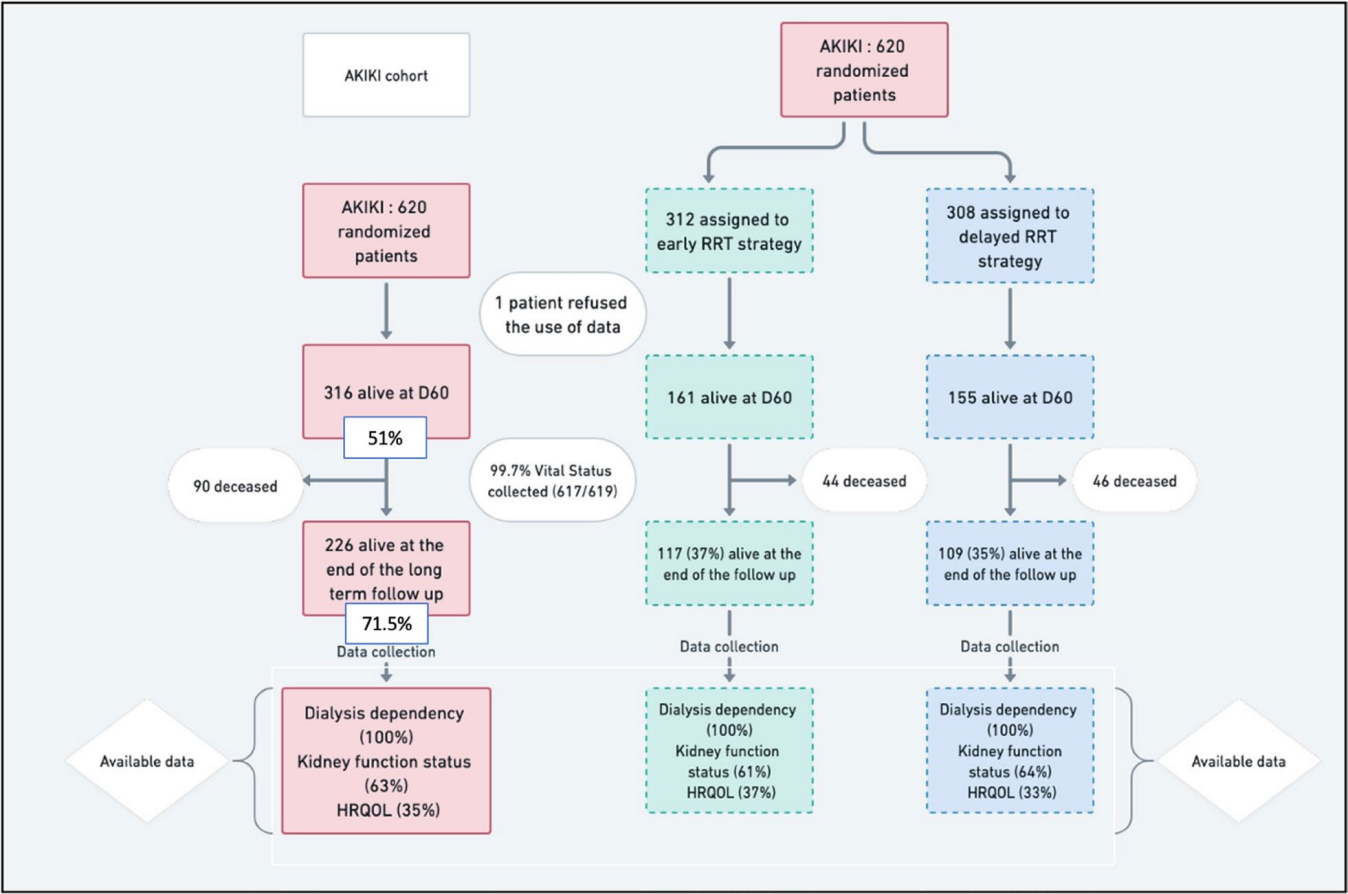
Study flowchart

Of the 619 patients included in the AKIKI trial [16], 316 (51%) survived for at least 60 days after randomization (Fig. 1). Fifty (16%) surviving patients had CKD at baseline. The Kaplan–Meier estimate of median follow-up after the AKI episode was 3.35 years (95% CI 2.72 to 3.16; IQR: 1.21 to 4.06). Baseline characteristics of patients are depicted in Table 1.

**Table 1:** Characteristics of the patients at baseline^a^

	AKIKI trial	Surviving patients 60 days after randomization			Surviving patients with Scr available beyond D90 after randomization
No of patients	*N* = 619	All *N* = 316	Early RRT strategy *N* = 161	Delayed RRT strategy *N* = 155	*N* = 175
Age—yr	66 ± 13.8	62.3 ± 14.7	60.8 ± 14.7	64 ± 14.7	62 ± 13
Sex—no. (%)					
Female	212(34)	110 (35)	55 (34)	55 (35)	60(34)
Male	407(66)	206 (65)	106(66)	100 (65)	115 (66)
Serum creatinine before ICU admission—mg/dl^b^	0.96 ± 0.28	0.95 ± 0.27	0.94 ± 0.25	0.96 ± 0.29	0.92 ± 0.23
Coexisting conditions—no. (%)					
Chronic kidney disease^c^	118 (19)	50 (16)	23(14)	27(17)	7(4)
Hypertension	328 (53)	153(48)	71(44)	82(53)	70 (40)
Diabetes mellitus	163 (26)	77 (24)	42(26)	35(23)	35(20)
Congestive heart failure	56 (9)	17 (5)	8 (5)	9(6)	3(2)
SAPS III at Enrollment^d^	73.1 ± 14.3	70.4 ± 14.8	70.8 ± 15.12	70 ± 14.05	66 ± 11
SOFA score at enrollment^e^	10.9 ± 3.2	10.3 ± 3	10.5 ± 3.11	10.16 ± 2.85	9.8 ± 2.6
Exposure to at least one nephrotoxic agent within two days before ICU—no./total no. (%)^g^					
All	389 (63)	178 (56)	93 (58)	85 (55)	92(53)
Intravenous contrast	137 (22)	70 (22)	34/93 (37)	36/85 (42)	35/92 (38)
Aminoglycoside	212(34)	95 (30)	52/93 (56)	43/85 (51)	30/92 (33)
Vancomycin	55 (9)	27 (8)	12/93 (13)	15/85 (18)	5/92 (5)
Septic shock^g^	413 (67)	167 (53)	80 (50)	87 (56)	43/92 (47)

^a^Plus–minus values are means ± SD. To convert values for creatinine to micromoles per liter, multiply by 88.4

^b^The serum creatinine concentration before ICU admission was either determined with the use of values measured in the 12 months preceding the ICU stay or was estimated

^c^Kidney function was reassessed with Cockcroft formula using available serum creatinine at baseline

^d^The Simplified Acute Physiology Score (SAPS) III ranges from 0 to 146, with higher scores indicating more severe disease and a higher risk of death

^e^The Sepsis-related Organ Failure Assessment (SOFA) score ranges from 0 to 24, with higher scores indicating more severe organ failure

^f^Some patients were exposed to more than one nephrotoxic agent

^g^Septic shock was defined as sepsis induced hypotension despite fluid resuscitation of at least 30 ml per kilogram of body weight of intravenous fluid administered within the period spanning the 4 h before and 4 h after initiation of vasopressor therapy

### Long-term outcomes

**Fig. 2:**
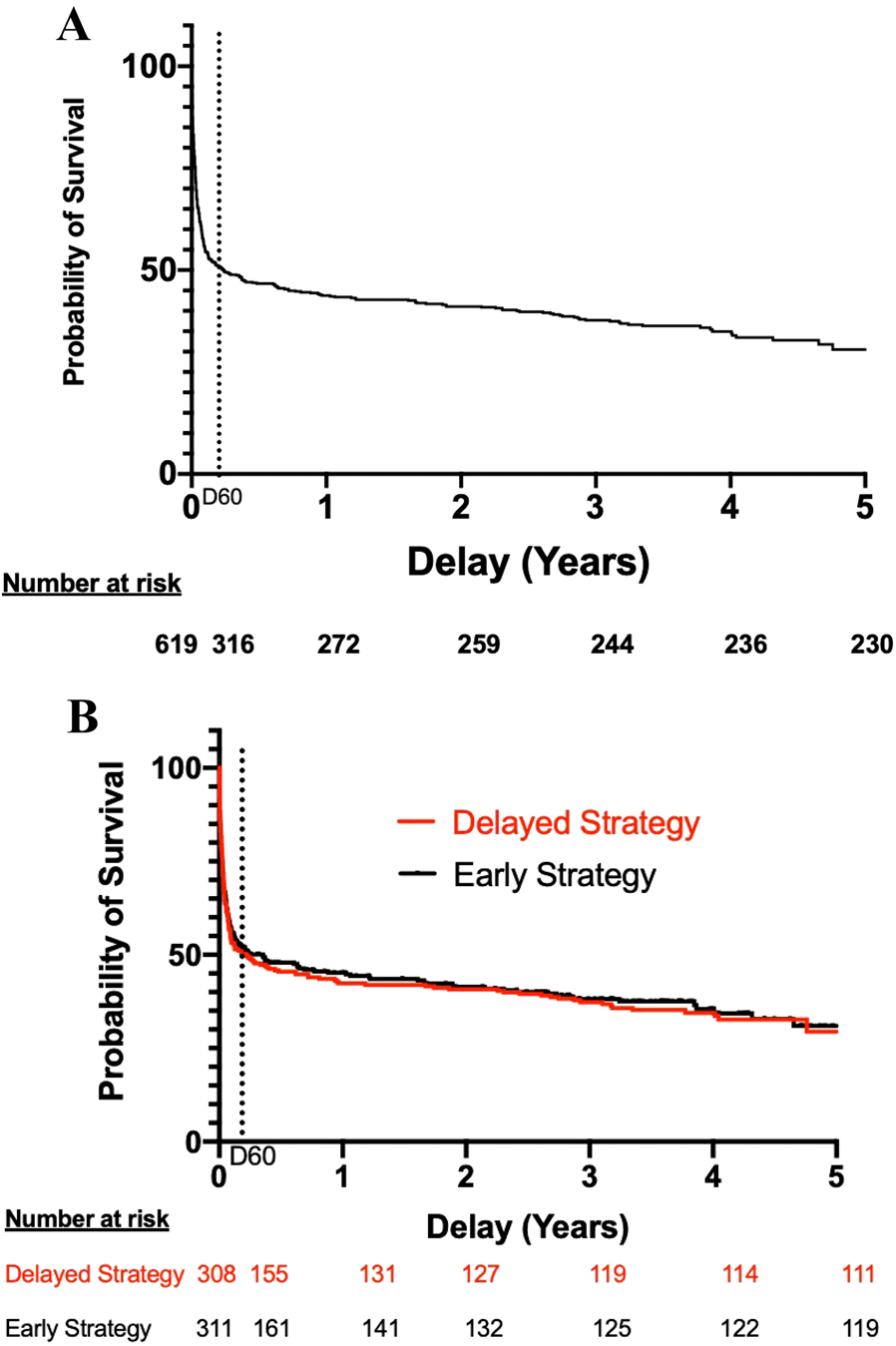
Probability of survival. Panel **A** shows Kaplan–Meier curves of the probability of survival for all study participants from randomization to end of extended follow-up. Panel **B** shows Kaplan–Meier curves of the probability of survival from randomization to end of extended follow-up, according to RRT initiation strategy group

**Fig. 3:**
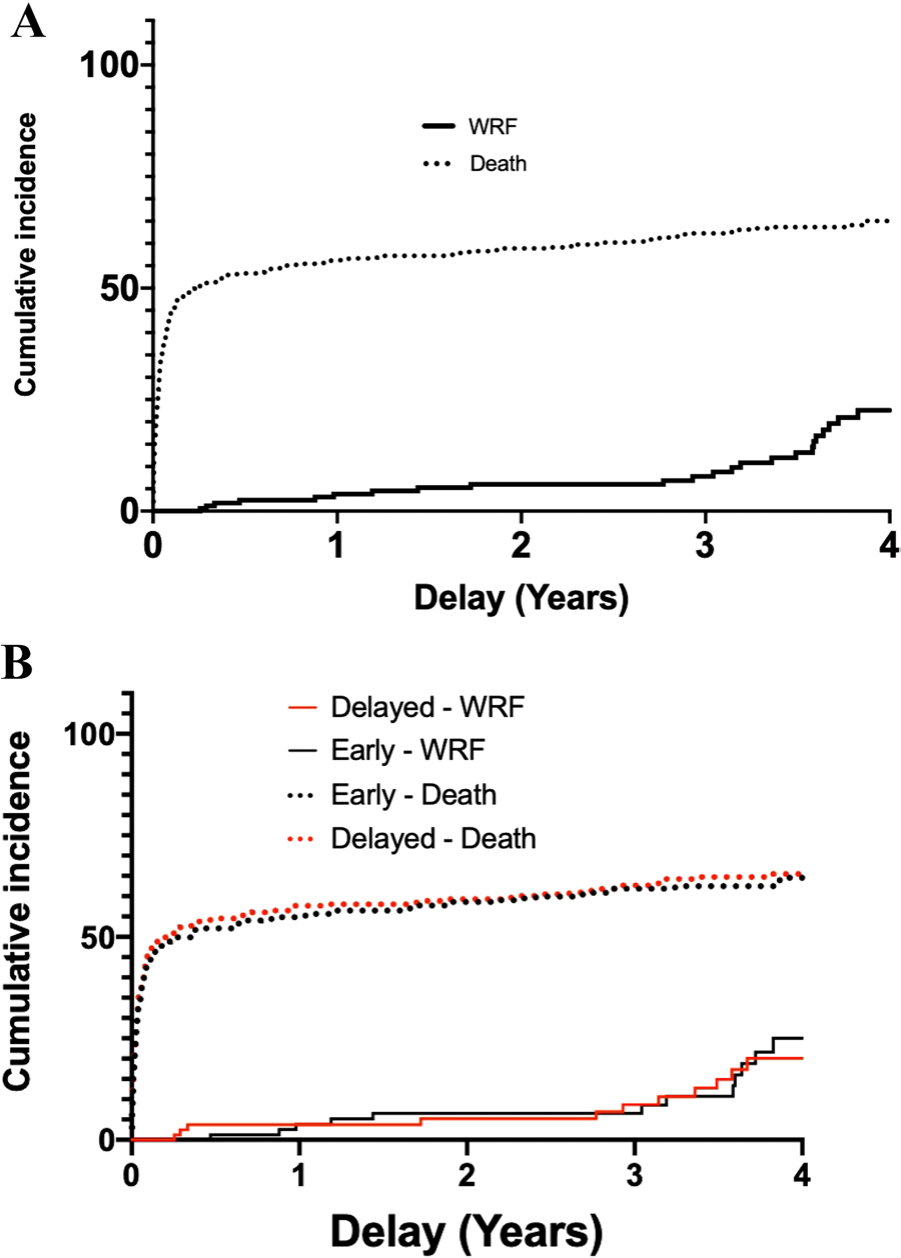
Cumulative incidence of WRF and death. Panel **A** shows cumulative incidence of WRF from D90 to end of extended follow-up for all study participants Panel **B** shows cumulative incidence of WRF and death from D90 to end of extended follow-up according to RRT initiation strategy group. *WRF* Worsening renal function (Defined for non-CKD patients at baseline by the occurrence of CKD and for CKD patients at baseline by the progression from a stage 3 to a stage 4 or 5)

**Fig. 4:**
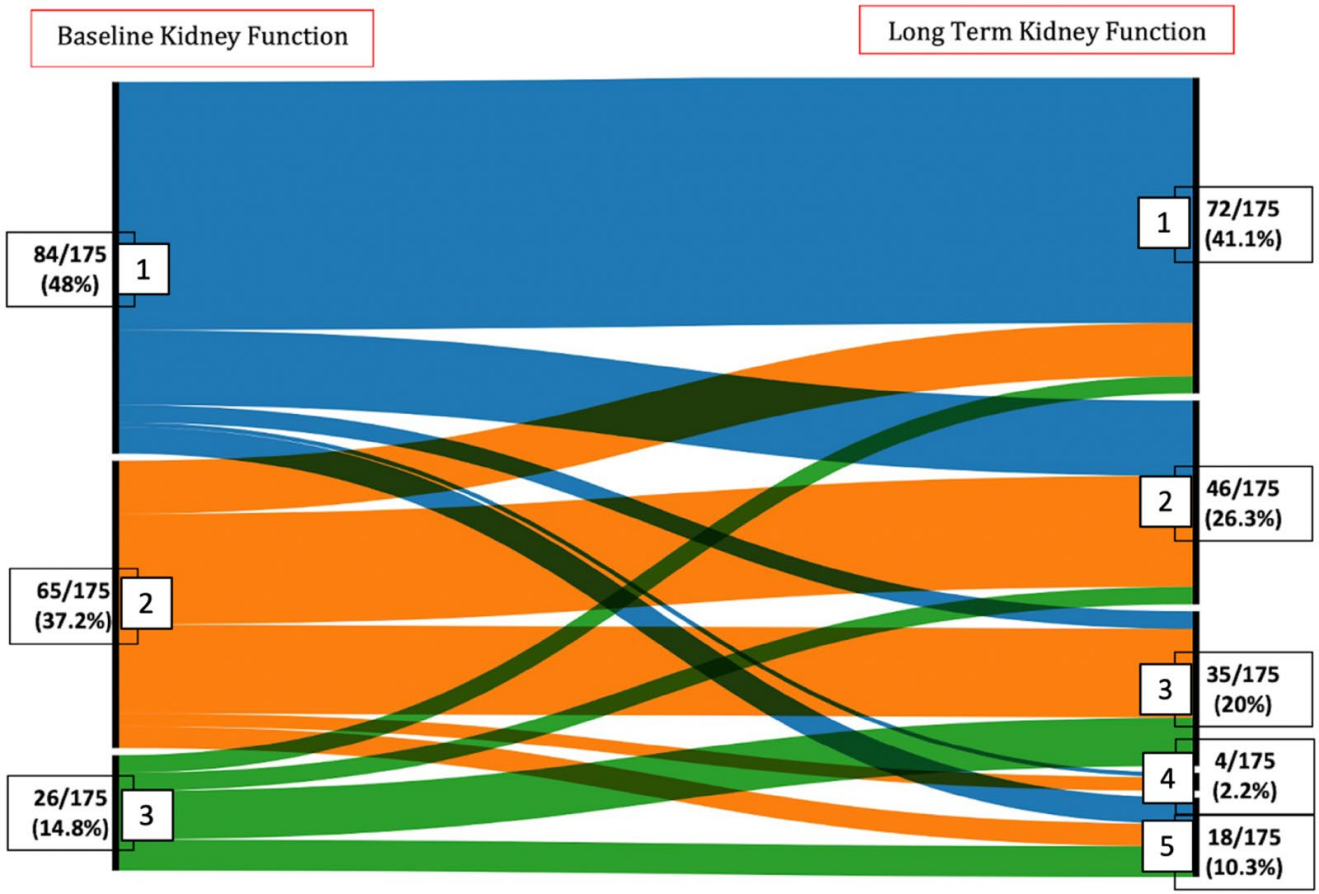
Alluvial diagram illustrating CKD stages evolution according to eGFR from baseline to end of follow-up. Each black bar represents an eGFR KDIGO stage at baseline (on the left of the diagram) and at the end of long-term follow-up (on the right of diagram). eGFR KDIGO stage: higher stages indicate more severe renal disease (see Additional file 1: Table S1 of the supplementary appendix).The height of the bars represents the proportion of patients. Alluvial diagram shows the evolution of the kidney function from baseline to long-term follow-up. The height of each stream represents the proportion of patient. Numbers and proportions of patients are presented next to each stage. Among these patients, 7 had CKD at baseline and eventually progressed from stage 3 to a higher stage of CKD, whereas 39 had normal renal function at baseline. Some patients had apparent eGFR improvement. This point is discussed in the text

- Survival
  Overall survival rate was 259/619 patients (41.8% (95% CI, 38 to 45.8)) and 244/619 (39.4% (95% CI 35.4 to 43.4)), at two and three years from inclusion, respectively. At the end of long-term follow-up, 226 patients were still alive accounting for an overall survival rate after day 60 of 226/316 (71.5%) and for a total of 226/619 (36.5%) of patients initially enrolled. Figure 2A shows the overall long-term survival for the 619 patients included in AKIKI trial. Survival did not differ according to the RRT initiation strategy allocated by randomization (Fig. 2B).
  Results of the univariate analysis with all screened variables are presented in supplementary appendix (Additional file 1: Table S2). Multivariate Cox models are summarized in Table 2. The only baseline variable that was statistically significant predictive of long-term mortality was age (HR 1.02, 95%CI 1.01 to 1.03).
- Renal outcomes
  There were no missing data for dialysis dependency and kidney transplantation. Values for SCr concentration beyond day 90 were available in 175 (63%) of 280 patients who survived after day 90. The median time between inclusion and latest SCr concentration assessment was 2.78 years (CI95% 1.58 to 3.14).
  The cumulative incidence of WRF was 15/175 (8.5% (CI95% 4.0–12.7)), and 36/175 (20.6% (CI 95% 13.0–28.3)) at three and four years after the severe AKI episode, respectively (Fig. 3A). At the end of follow-up, 46/175 patients (26.2%) presented WRF. Among these patients, 7 had CKD at baseline and eventually progressed from stage 3 to a higher stage of CKD, whereas 39 had normal renal function at baseline. The occurrence of WRF did not differ according to RRT initiation strategy (Fig. 3B). In the univariate Fine and Gray analysis, no variable was associated with the occurrence of WRF (Table 3).
  The evolution of eGFR between baseline and long-term follow-up of surviving patients is presented in Fig. 4.
  Among the 280 patients who survived after day 90, six remained dialysis-dependent after their ICU stay. Fifteen patients eventually needed chronic dialysis (5%), after a median of 2.3 months (0 to 42) (after day 90). No patient received a kidney transplant.
- HRQOL
  Among long-term survivors, 80/226 (35%) answered the EQ-5D questionnaire. The median index value was 0.67 (IQR 0.40 to 1.00). The violin plot of index values is presented in supplementary appendix (Additional file 1: Figure S1). The values for the 5 dimensions of the questionnaire are presented in the supplementary appendix (Additional file 1: Figure S2).
  Inclusion of deceased patients in the calculation yielded a median index of 0.39 (IQR 0.20 to 0.81) (Table 4). The RRT initiation strategy had no effect on results of HRQOL (Table 4).

**Table 2:** Cox multivariate model for long-term mortality from randomization

Variable	Comparator	Hazard ratio (95% CI)	*P* value
Strategy	Early	1.01 (0.82–1.23)	0.9
SAPS III	One unit increase	1 (0.99–1.014)	0.12
CCF		1.28 (0.91–1.8)	0.15
CKD		1.04 (0.80–1.36)	0.74
Diabetes		0.93 (0.74–1.18)	0.56
Age	One unit increase	1.02 (1.01–1.03)	p < 0.01
Sex	Male	1.08 (0.86–1.34)	0.51
Hypertension		0.92 (0.73–1.15)	0.45

*SAPSIII* Simplified Acute Physiology *Score 3 CCF* chronic cardiac failure *CKD* chronic kidney disease

**Table 3:** Univariable analysis of subdistribution hazard ratios for factors associated with WRF

Variable	Comparator	Sub-hazard ratio (95% CI)	*P* value
Strategy	Early vs delayed	1.34 (0.74–2.43)	0.33
SAPS III	One unit increase	1 (0.97–1.04)	0.93
CCF		1.34 (0.47–5.27)	0.47
CKD		1.34 (0.62–2.89)	0.46
Diabetes		1.17(0.61–2.26)	0.64
Age	One unit increase	1.02 (0.99–1.04)	0.14
Sex		0.73 (0.40–1.33)	0.31

Analysis led on the 175 patients with available eGFR after D90

*SAPSIII* Simplified Acute Physiology *Score 3 CCF* chronic cardiac failure *CKD* chronic kidney disease

**Table 4:** Quality of life

Variable	All patients	Early RRT strategy (95% CI)	Delayed RRT strategy (95% CI)	*P* value
EQ5D-5L (survivors at end of follow-up)a	0.67 (0.40–1.00)	0.71 (0.51–1.00)	0.64 (0.50–1.00)	0.32
EQ5D-5L (patients since D60)^b^	0.39(0.20–0.81)	0.35 (0.22–0.82)	0.41 (0.20–0.80)	0.70

Analysis of the survivors was led on the 80 patients who responded to the questionnaire. Analysis of patients since D60 was led on 170 patients (90 patients who died after D60 and 80 patients who responded the questionnaire). Index values are a summary of the 5 dimensions described in the manuscript

^a^All Index Values represented are medians. Interquartile ranges are in ()

^b^Including deceased patients. Death was also treated as an event because the EQ-5D index value of deceased patients is normally considered to be 0

## Discussion

In this prospective study, 619 critically ill patients with severe AKI were followed for a median time of 3.35 years and for a maximum of 7.39 years. As previously reported, D60 mortality was 49% (95% IC 43 to 54.2) [16]. Death eventually occurred in 28.5% of patients who had survived until day 60 in the initial study. Then, overall survival at 3 years from inclusion was 39.4% (95% CI 35.4 to 43.4). During long-term follow-up, WRF developed in more than a quarter of patients. The occurrence of WRF was noticeable in the first months of follow-up and increased substantially 3 years after the severe AKI episode. However, the proportion of patients needing chronic dialysis was small (5%) and no patient received a kidney transplant. The alluvial diagram (Fig. 4) shows that end stage renal disease (ESRD) was the final path in all patients with CKD at baseline who suffered from worsening of their renal function after the episode of AKI. Then, such patients should be followed very carefully since worsening of renal function can be dramatically rapid. The HRQOL was noticeably impaired in this population. Finally, long-term survival, kidney function and quality of life did not significantly differ according to the RRT initiation strategy.

As could be anticipated, mortality was high in the first 60 days (303/619, 49%) [16] but less so during long-term follow-up. The RENAL trial reported comparable figures (long-term survival of 38% with most deaths occurring in the first 3 months [24]). These observations are consistent with the hypothesis that comorbidities become the most impacting entity on life expectancy after recovery from an episode of AKI [25]. However, the RENAL study included only patients who received RRT, excluding patients with severe AKI who died or recovered before initiation of RRT, whereas ours concerns all patients with severe AKI allocated to an early or delayed RRT initiation strategy (which allowed a substantial proportion of patients to escape RRT).

A high proportion (more than one-quarter) of patients experienced WRF during follow-up in our study. These figures differ from those reported in a study on cardiac surgery patients [26]. Indeed, in that study only 5.7% of patients who experienced AKI and survived developed CKD during follow-up (that study did not provide a median time of follow-up). However, that study comprised a population with different stages (KDIGO 1 to 3) of reversible AKI, whereas ours included only patients with AKI KDIGO stage 3. A recent prospective study (ASSESS-AKI) [27] found consistent results with our study but included only 97 patients with AKI stage 3 on a total of 769 patients (561 had stage 1 AKI and 111 stage 2). Our study focused on a large (*n* = 619) and homogeneous population of AKI stage 3. Indeed, the risk of developing long-term renal consequences varies probably with the severity of renal insult as well as with the global severity of patients. The small proportion of patients who eventually needed chronic dialysis (5%) in our study is, however, comparable to most studies which provide a follow-up of more than 3 years [28–30]. However, another study reported a higher proportion of chronic dialysis (25%) after an episode of AKI in a population of critically ill patients [31]. This difference might be explained by a high proportion (28%) of patients with CKD before the index hospitalization admission in that study. In contrast, only 19% of patients had preexisting CKD in our study.

A noticeable caveat of all studies, including ours, reporting renal function outcome stems from the fact eGFR may be overestimated by Cockcroft formula at the end of ICU-stay in patients because of muscular loss [32]. This may be responsible for some of our patients moving up into a lower stage of CKD in our study (Fig. 4).

Another important issue is related to the influence of the RRT initiation strategy during AKI. Since three recent large multicenter studies did not show any difference of short-term (60 to 90  days) outcome according to RRT initiation strategy, delaying RRT initiation in patients with severe AKI and no life-threatening complication will probably become standard of care [33]. In such conditions, it is important to evaluate long-term outcomes and particularly whether the initiation strategy affects any of these outcomes. This issue seems controversial at present. Indeed, the follow-up of patients included in the ELAIN trial [34] for one year was the first study to report mortality data at a distance of an initial episode of AKI, according to RRT initiation strategy. Findings indicated that an early initiation of RRT during AKI was associated with a considerably lower mortality rate after 90 days in the early strategy group as compared with the delayed one (19% additional versus 39% in the delayed group, p 0.005). In that study, the time difference of RRT initiation between the two strategies was less than 24 h. Authors do not offer explanation for the finding that such small difference produced very important effects on long- term outcomes. Several features of the initial ELAIN study differ with ours. ELAIN was a single-center study that included mainly post-cardiac surgery patients with a high proportion of fluid overload and worsening pulmonary edema [35], whereas the present one was a multicenter study that included patients with various medical conditions and a high proportion with sepsis or septic shock. In addition, ELAIN study reported a noticeable difference in short-term (90 days) survival between patients allocated to an early RRT strategy (39%) and those allocated to a delayed strategy (55%, *p* < 0.03), whereas the 3 above-mentioned large multicenter RCTs (accounting for a total of 4034 patients) as well as a large (1879 patients) individual patient-data meta-analysis did not report difference in mortality at D60 or 90 according to RRT initiation strategy.

In fact, the risk of renal sequelae might be higher with an early strategy RRT initiation strategy during AKI for various reasons [33]. Indeed, delayed renal function recovery was observed with such strategy in both AKIKI [16] and STARRT-AKI study [36]. These findings might suggest an association between an early RRT strategy and a risk for CKD [37]. Our present study was unable to confirm this hypothesis. In summary, the discrepancy concerning long-term outcome between the ELAIN study and ours is unexplained at present and underlines the importance of gathering data from more studies before a definite conclusion can be drawn.

Multiple evidence suggests that AKI portends long-term risks although not reported in this study (cardiovascular events, sepsis, fracture risk, CKD and chronic dialysis) making a HRQOL assessment interesting to analyze [3, 38, 39]. In our study, HRQL was noticeably impaired among survivors of AKI with a score comparable to what was found in a systematic review published in 2016 [40]. The initial RRT strategy had no effect on the quality of life.

The main strength of the present study is the extended follow-up of more than 3 years, with some patients followed for more than 7 years and the provision of long-term follow-up according to initial RRT timing strategy in critically ill patients. Results suggest that delaying RRT for critically ill patients in the absence of life-threatening condition does not affect long-term outcomes, in addition to be safe on a short-term basis. The long duration of follow-up allowed to identify definitive WRF. Indeed, a previous study demonstrated that it takes time (up to one year) in order for patients to reach their highest eGFR after an episode of AKI [28], leading to overestimation of WRF if follow-up is too short.

Our study suffers from some limitations. First, a substantial proportion of data on kidney function and HRQOL was not available at the time of follow-up leading to missing data. Since data were missed completely at random [41] (patients and families contact information missing at admission), bias in data collection is unlikely. However, missing data may have affected the precision of our results; therefore, caution is advised in drawing inferences from these findings. This issue is common to all studies assessing long-term renal follow-up [42] and the proportion of missing data in our study is comparable to others [24, 40]. Second, we did not perform a population-based study with non-AKI matched cohort. Then, we do not provide hazard ratio for risk of death, WRF and HRQOL impairment. Third, we did not assess urinary sediment or proteinuria although it is an important information for CKD staging in KDIGO guidelines [15, 43]. Nevertheless, studies reporting these data are often flawed by the non-separation of preexisting proteinuria and hematuria from newly discovered cases after admission [44]. Since our study included more than a quarter of patients with diabetes, it is likely that it would have suffered the same pitfall.

## Conclusion

The present study documents long-term outcomes of critically ill patients with severe AKI accurately. Although death rate markedly abates after the first 2 months, death toll remains noticeable and a substantial proportion of survivors progress to CKD and have HRQOL impairment more than 3 years after. These outcomes did not differ according to RRT initiation strategy.

## Supplementary Information

**Additional file 1: Table S1.** Current Chronic kidney disease Nomenclature used by KDIGO. **Table S2.** Univariate analysis of mortality predictors. **Figure S1.** Violin plot HRQL. **Figure S2**. EQ5D five dimensions of health in SALTO according to RRT strategy group. **Figure S3.** Schoenfeld residuals for proportional hazards assumption.

## Data Availability

The datasets used and/or analyzed during the current study are available from the corresponding author on reasonable request.
