# Supplementary material for: Long-term outcomes after severe acute kidney injury in critically ill patients: the SALTO study
Source: Ann Intensive Care. 2023 Mar 13;13:18. doi: 10.1186/s13613-023-01108-x (PMC10008759; doi:10.1186/s13613-023-01108-x)
Supplement: Supplementary file 1 — Additional file 1: Table S1. Current Chronic kidney disease Nomenclature used by KDIGO. Table S2. Univariate analysis of mortality predictors. Figure S1. Violin plot HRQL. Figure S2. EQ5D five dimensions of health in SALTO according to RRT strategy group. Figure S3. Schoenfeld residuals for proportional hazards assumption. [file 13613_2023_1108_MOESM1_ESM.docx]

**Additional file appendix**

TABLE OF CONTENTS OF SUPPLEMENTAL ELECTRONIC MATERIAL

**INCLUSION CRITERIA**

**TABLES**

**Table S1
Table S2**

**FIGURES**

**Figure S1
Figure S2**

**INCLUSION CRITERIA**

Eligible patients were adults (18 years of age or older) admitted to the ICU with AKI
compatible with the diagnosis of acute tubular necrosis in a context of ischemic or toxic
aggression and receiving invasive mechanical ventilation and/or catecholamine infusion
(epinephrine or norepinephrine). To be randomized, patients should have AKI stage 3 of
KDIGO classification defined by at least one of the following criteria: serum creatinine
concentration of more than 4 mg/dl (354 μmol/liter) or greater than 3 times the baseline
creatinine level, anuria (urine output of 100 ml/day or less) for more than 12 hours, oliguria
(urine output below 0.3 ml/kg/h or below 500 ml/day) for more than 24 hours.
Randomization and initiation of treatment (RRT or conservative treatment) were mandatory
within six hours after fulfillment of the final inclusion criteria.

The non-inclusion criteria were:

- Presence of one of the following conditions: blood urea nitrogen of more than 112
md/dl (40 mmol/liter), serum potassium concentration of more than 6 mmol/liter or
more than 5.5 mmol/liter persisting despite medical treatment (bicarbonate and/or
glucose-insulin infusion), pH below 7.15 in a context of pure metabolic acidosis
(PaCO2 below 35 mmHg) or in a context of mixed acidosis with PaCO2 of 50 mmHg
or more without possibility of increasing alveolar ventilation, acute pulmonary edema
due to fluid overload responsible for severe hypoxemia requiring oxygen flow rate of
more than 5 L/min to maintain an SpO2 of more than 95% or an FiO2 greater than 50%
in patients already on invasive or non-invasive mechanical ventilation and despite
diuretic therapy.
-Pre-existing severe chronic renal failure (defined by a creatinine clearance < 30 ml/min)
-Patients already included in the study
-Patients with inclusion criteria already present for more than 5 hours (to avoid delayed
inclusions)
-AKI caused by urinary tract obstruction or renal vessel obstruction or tumour lysis
syndrome or thrombotic microangiopathy or acute glomerulopathy
-Poisoning by a dialyzable agent
-Child C liver cirrhosis
-Cardiac arrest without awakening
-Moribund state (patient likely to die within 24h)
-Patient having already received RRT for the current episode of AKI
-Extracorporeal lung or circulatory assistance
-Patients included in another clinical study of a RRT technique.
-Renal transplant
-Treatment limitation (withholding or withdrawal)

**Table S1. Current Chronic kidney disease Nomenclature used by KDIGO**


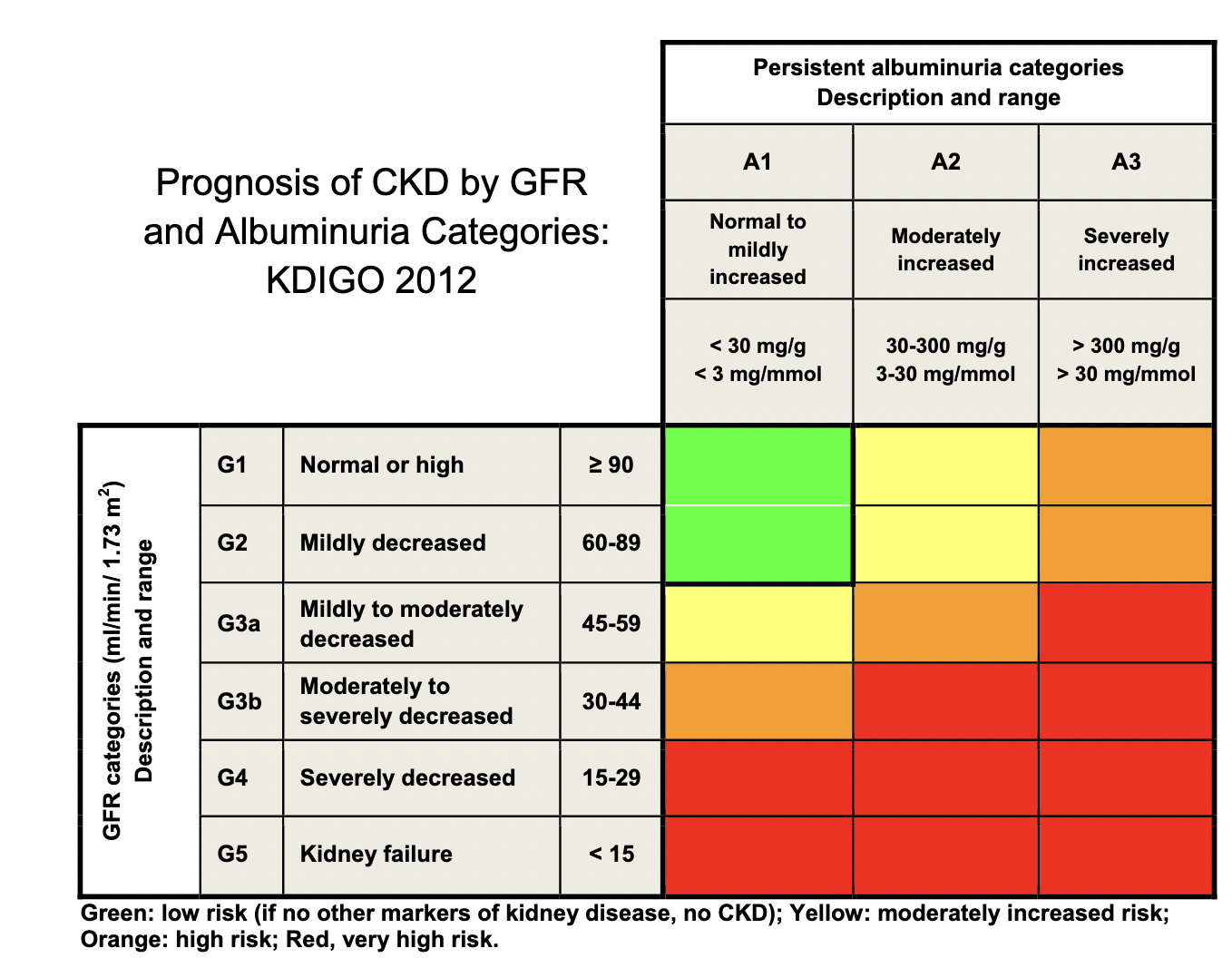


**Table S2 Univariate analysis of mortality predictors**

| Variable | Comparator | Hazard Ratio (95% CI) | P value |
| --- | --- | --- | --- |
| Strategy | Early | 1.03 (0.84-1.26) | 0.80 |
| SAPS III | One unit increase | 1.01 (1.003-1.018) | <0.01 |
| CCF |  | 1. 36 (0.99-1.87) | 0.06 |
| CKD |  | 1.33(1.045-1.68) | 0.02 |
| Diabetes |  | 1.09(0.87-1.36) | 0.40 |
| Age | One unit increase | 1.021 (1.013-1.028) | <0.01 |
| Sex |  | 1.075 (0.87-1.33) | 0.50 |
| Hypertension |  | 1.12 (0.92-1.37) | 0.20 |

SAPSIII: Simplified Acute Physiology score 3

CCF : Chronic Cardiac Failure

CKD : Chronic Kidney Disease

**Figure S 1 Violin plot HRQL**


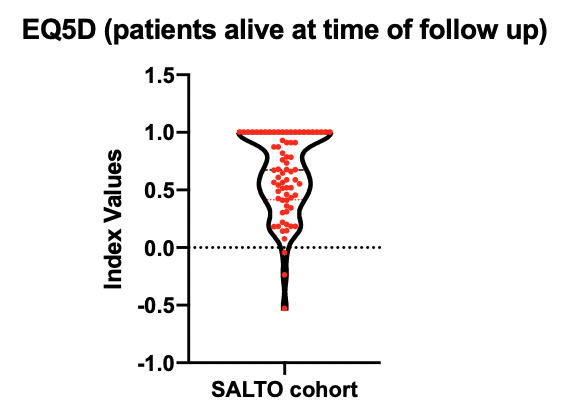


**Figure S 2 EQ5D five dimensions of health in SALTO according to RRT strategy group**

Mobility

Usual activities

Anxiety/depression

Pain/Discomfort

Self care

**Index values :**

**Early Strategy : 0.68**

**Delayed Strategy : 0.60**

P=0.37


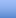
 Early strategy

Delayed strategy


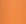


**EQ5D 5L**

**Figure S 3 Schoenfeld residuals for proportional hazards assumption**


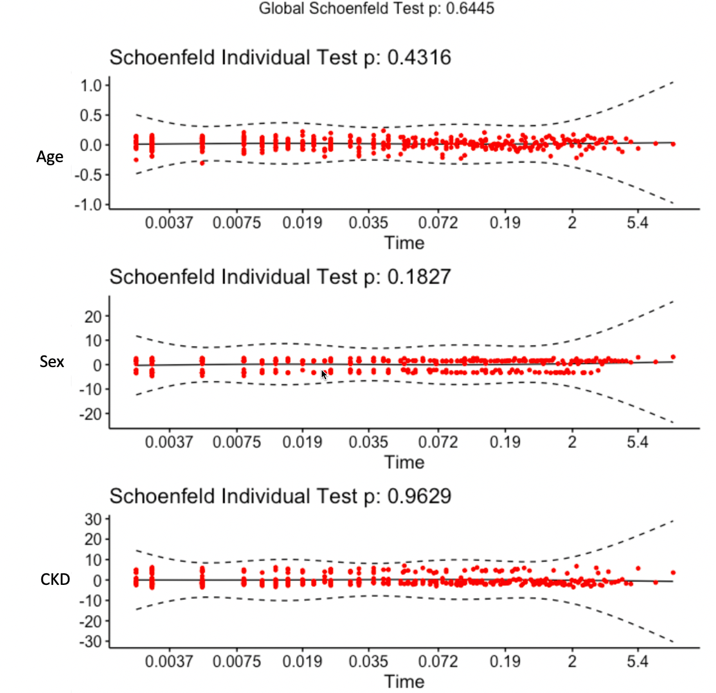

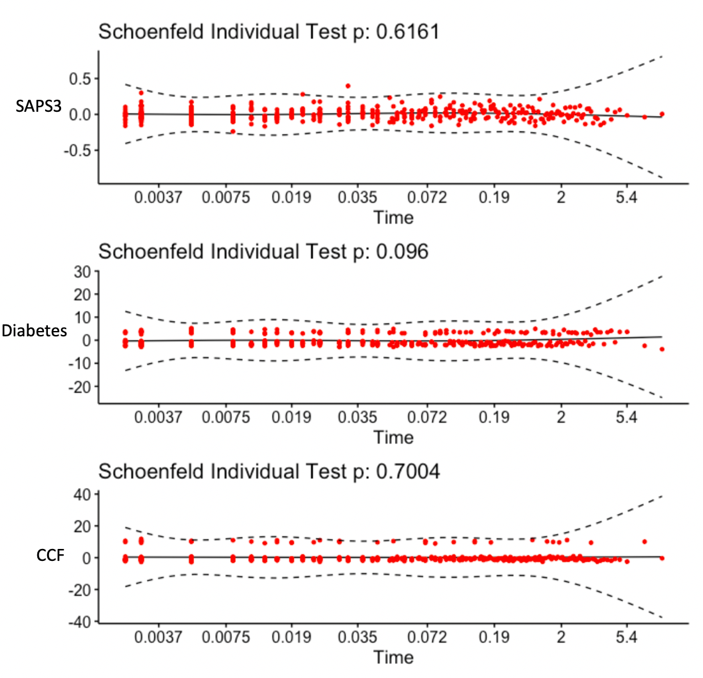


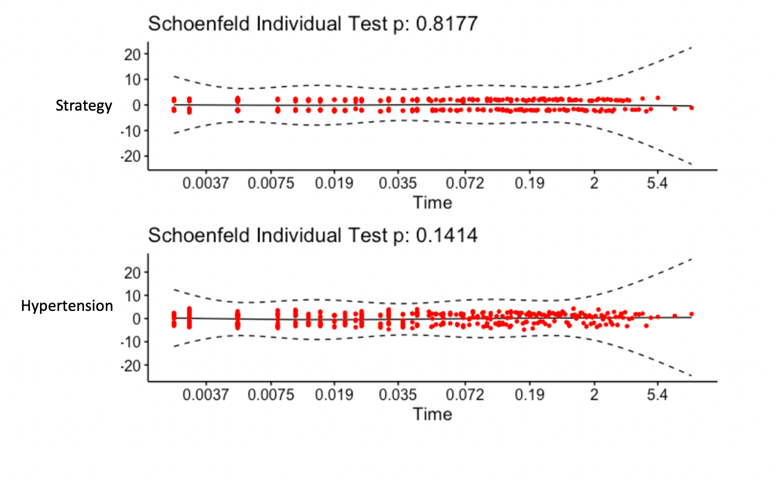


SAPS3: Simplified Acute Physiology score 3

CCF : Chronic Cardiac Failure

CKD : Chronic Kidney Disease
